# Domesticating Cancer: An Evolutionary Strategy in the War on Cancer

**DOI:** 10.3389/fonc.2017.00304

**Published:** 2017-12-07

**Authors:** Gustav van Niekerk, Theo Nell, Anna-Mart Engelbrecht

**Affiliations:** ^1^Department of Physiological Sciences, Stellenbosch University, Stellenbosch, South Africa

**Keywords:** synthetic lethality, auxotrophy, cancer, evolution, chemoresistance

## Abstract

Since cancer shares the same molecular machinery as the host, most therapeutic interventions that aim to target cancer would inadvertently also adversely affect the host. In addition, cancer continuously evolves, streamlining its host-derived genome for a new single-celled existence. In particular, short-term clinical success observed with most antineoplastic therapies directly relate to the fact that cancer is constantly evolving. However, the clonal evolution of cancer occasionally also render cancer cells uniquely susceptible to therapeutic interventions, as is exemplified by the clinical relevance of synthetic lethality. Synthetic lethality describes a situation where the simultaneous loss of function in two genes results in lethality, but where a loss of function in either single gene is tolerated. This observation suggests that the evolution of cancer, usually seen as a major clinical challenge, may also afford a key opportunity in lowering on-target toxicities accosted with chemotherapy. As an example, by subjecting cancer to specific selection regimes, cancer can in effect be placed on evolutionary trajectories leading to the development of “targetable” phenotypes such as synthetic lethal interactions. However, such a selection regime would have to overcome a range of obstacles such as on-target toxicity and the selection of an evolvable trait. Since the majority of cancer evolution manifests as a loss of function, we suggest that the induction of auxotrophic phenotypes (i.e., where an organism lose the ability to synthesize specific organic compounds required for growth and thus become dependent on it from dietary sources) may represent an attractive therapeutic option. As an example, animals can obtain vitamin C either by *de novo* synthesis or from their diet. However, since the maintenance of synthetic pathways is costly, such pathways are often lost if no longer necessary, resulting in the organism being auxotrophic toward the dietary compound. Similarly, increasing the maintenance cost of a redundant pathway in cancer cells is likely to select for clones that have lost such a redundant pathway. Inhibition of a pathway, while supporting the activity of a compensating pathway, may thus induce auxotrophism in cancer cells but not in genomic stable host cells.

## Introduction

The fact that cancer is derived from the host’s own tissue possess a major challenge for the development of effective therapies. Since cancer shares the same molecular machinery as host, most therapeutic interventions aimed at targeting cancer would inadvertently also adversely affect the host tissue. Indeed, the small therapeutic window of most chemotherapeutic agents directly relates to the on-target toxicity associated with chemotherapy. The problem is compounded by the fact that the host represents a complex system of interdependent organs and tissues systems. Consequently, patient tolerance toward therapeutic agents is usually limited by the tissue exhibiting greatest sensitivity toward the chemotherapeutic intervention. The evolvability of cancer represents another clinical challenge. In a few cases, targeted therapy induces cancer remission with comparatively little side effects. Yet, it has been noted that such therapies successes are usually short lived and only extend survival by a few months in advanced cancer (1). Indeed, in most cases, therapeutic intervention often results in a significant decline in tumor size only for cancer to re-emerge as chemo-resistant strains (2–5). Thus, the fact that cancer shares the same cellular mechanism and components as the host, in conjunction with the evolvability of cancer, remains a major challenge in the development of a cure for cancer.

Thus, these challenges explain why, despite the tremendous advances made in understanding the biology of cancer, in the majority of cases, we have not won the war on cancer yet (6). Accordingly, some have proposed that we rethink the problem of cancer in an attempt to develop novel therapeutic interventions (7). In this regard, the application of ecological principals in managing cancer has similarly gained much attention (8–11). Indeed, many concepts used to describe ecological interactions are easily transferable to an oncological context. As an example, predation (immune surveillance), niche construction (tumor micro-environment), and concepts such as fitness (proliferative potential of cancer cells in a given environment) all encompass cancer relevant traits (9). Furthermore, an ecological perspective addresses the evolvability of cancer. As an example, it has been pointed out that under certain conditions, chemoresistance to one compound may result from a set of mutations that render cancer cells “collateral sensitive” toward another agent (12). Similarly, instead of aggressively applying therapies aimed at completely eradicating cancer, low-dose chemotherapy aimed at reducing tumor load while sparing chemosensitive cancer cells may hold an advantage as these cells compete with drug-resistant strains, thus limiting the growth of these strains (11). An ecooncological view thus opens novel therapeutic strategies to the challenges posed by drug resistance.

Although these “ecology-based” therapies address the evolution of cancer, we suggest that cancer evolution may also provide clinical opportunities. In particular, carefully chosen selective pressures could potentially be used to direct the evolution of traits that render cancer cells more sensitive to the subsequent therapeutic interventions, thus lowering on-target toxicity. However, the evolution of cancer cells differs in a number of key aspects from evolutionary processes associated with other organisms, presenting opportunities, as well as challenges in directing the evolution of cancer. These issues are briefly reviewed after which we outline a hypothetical approach for directing the evolution of cancer cells.

## Cancer Evolution

Cancer cell heterogeneity has emerged as a key factor in the development of chemo-resistance (13, 14). Tumor heterogeneity results not only from phenotypic plasticity (15) but also arises due to the numerous mutations that drive the evolution of drug resistance (16). Indeed, it has been argued that the astonishing number of mutations observed in many tumors (2, 16–19) cannot be explained by normal background mutational rate and that certain cancers possess a “mutator phenotype” (20, 21). Mechanistically, an elevated rate of mutations observed in cancer cells may relate to increasing aneuploidy (22) and to alterations in the DNA damage response, which decrease the ability of cancer cells to correct DNA damage (23). In addition, various chemotherapeutic agents are mutagenic (24), thus potentially enhancing the mutation rate. Furthermore, the ability of cancer to evolve (e.g., exhibit a mutator phenotype) might be more than an accidental trait: both theoretical models and simple single-cell systems have demonstrated that, in an environment that is rapidly changing, the ability to evolve may itself be a trait under Darwinian selection (25). Since chemotherapy represents a major shift in the tumor “ecosystem,” therapeutic interventions may in fact select for an “evolvable” phenotype. These observations thus explain the highly evolvable nature of cancer cells that invariably lead to the development of drug resistance.

The clonal evolution of cancer cells differs from typical evolutionary processes in several ways. First, since cancer arises with most of the host genome intact, cancer cells are pre-equipped with various genomic protocols for subverting host systems. This is exemplified by the observation that tumors typically re-enact a transcriptional profile observed during wound healing (26). Similarly, invasive cancer cells re-enact transcriptional profiles associated with placentation (27, 28). These observations demonstrate how cancer evolve not only by the generation of novel functionalities but also by “rediscovering” pre-existing genomic content usually not activated in untransformed tissue. In addition, the evolution of cancer is also constrained in certain unique ways. As an example, the fact that cancer only persists within the life span of the patient limit the range of phenotypes, which can be selected for (14, 29). Furthermore, cancer cells reproduce asexual and exhibit a very high mutation rate with genomic instability, and most of the mutations tend to involve a loss of gene function, rather than the evolution of novel gene functions. In addition, there is a bias in the kind of genes that are most often mutated (e.g., loss of pro-apoptotic proteins such as p53). In fact, it has also become apparent that not all genes are equally evolvable: in cancer, inactive genes tend to exhibit a higher mutation burden, probably as a result of reduced transcription-coupled repair processes (30). Collectively, these observations suggest that cancer is highly evolvable, but that the mechanism underlying cancer evolution is distinct and largely associated with the exploitation and rediscovering of a pre-existing genome.

## Exploiting Loss of Function: Synthetic Lethality

**Figure 1:**
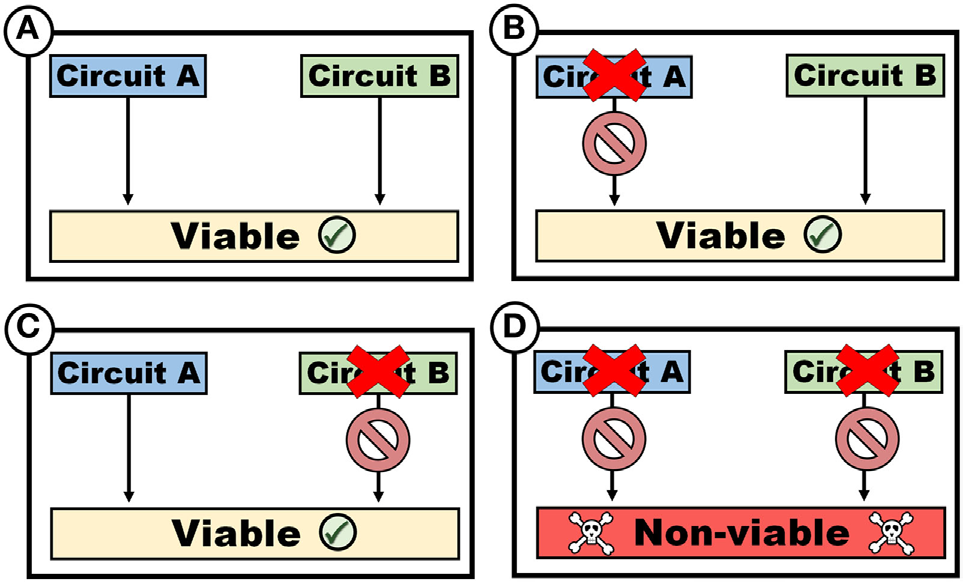
Synthetic lethality. Two circuits, which may represent variably functions such as biosynthetic pathways or cellular functions such as genomic repair mechanisms, are synthetically lethal if viability is maintained despite the loss of either single circuits [**(B)** or **(C)**], but not both **(D)**.

The evolution of cancer also leads to the development of genomic dependencies that render cancer cells more vulnerable to therapeutic intervention, a situation well exemplified by synthetic lethality. Consider a system that consists of two effector pathways circuit A and B within a cell, which operates independent of each other, in a manner that render them functionally redundant (Figure 1). As an example, A may represent a mechanism to import a factor required (e.g., an amino acid transporter), whereas B represents a pathway associated with the *de novo* synthesis of a required factor (e.g., synthesis of an amino acid). If both circuits are operational (Figure 1A), cells are viable. Similarly, if A is defective (Figure 1B), B can compensate, thus ensuring cell survival. Conversely, despite inactivation of B, cells remain viable as a result of the compensatory effect of A (Figure 1C). If, however, both A and B are inactivated, cell viability is compromised (Figure 1D). In such a system, A and B are synthetically lethal, since a loss of either A or B is tolerated, whereas the simultaneous loss of both A and B are not.

Synthetic lethality can be employed to elucidate the role of genes and map gene–gene interactions and has also gained interest in context of cancer therapy (31, 32). As an example, CRISPR-based screens of cancer cells have been used to identify synthetic lethal interactions between genes, thus exposing potential therapeutic targets, and has also revealed the functions of previously uncharacterized proteins (33). In fact, synthetic lethality interactions have also been shown to be of clinical relevance: cancer cells bearing mutations in *BRCA1* or *BRCA2*, which play a key role in the repair of double-strand DNA breaks, are susceptible to inhibitors of the enzyme poly ADP ribose polymerase, which also plays a role in repairing DNA lesions (34).

The implementation of synthetic lethality within an oncological context is dependent on the ability of cancer cells to evolve. In particular, the loss of function renders cancer cells dependent on compensatory genes. Although synthetic lethality has thus far been opportunistically exploited, we argue that cancer cells might be placed on evolutionary trajectories that would select for functional dependencies similar to synthetic lethality. However, a selection regime should exhibit certain properties. First, the evolution of a synthetically lethal phenotype must be an evolvable trait and also be responsive toward a clinically applied evolutionary pressure. Second, the selection regime used to direct the evolution of a synthetically lethal phenotype should be well tolerated and not be associated with severe side effects. Here, it is argued that the induction of an auxotrophic phenotype might be ideal, as such an approach can fulfill these requirements.

## Auxotrophism as a Form of Synthetic Lethality

Auxotrophy refers to the reliance of an organism on a biological compound for appropriate growth and development, but which cannot be synthesized by the organism itself. As an example, many vertebrates possess both effective mechanisms for acquiring ascorbic acid (vitamin C) from the diet and pathways for its *de novo* synthesis. In contrast, certain animals (e.g., humans, guinea pigs, certain bats and fish) have lost this ability to synthesize ascorbic acid—these animals exhibit vitamin C auxotrophy. The independent loss of capacity to synthesize vitamin C in numerous species with a diet high in vitamin C (35) may be explained by the fact that hydrogen peroxide formation during vitamin C synthesis (36) represents a cost for maintaining this pathway. In animals consuming a diet rich in vitamin C, the loss of biosynthetic capacity thus would be advantageous, thus explaining the loss of synthetic capacity in such animals (37).

Evidence suggests that cancer cells may similarly evolve auxotrophic phenotypes. As an example, certain cancers exhibit auxotrophy toward arginine as a result of reduced expression of argininosuccinate synthase (38, 39). In these cancerous cells, prolonged arginine starvation induces a form of autophagic cell death (40). In fact, recent results from a phase II multicenter randomized clinical trial demonstrated that arginine deprivation with pegylated arginine deaminase increased the progression-free survival of cancer patients (41). Similarly, Kung et al. (42) recently described a small molecule activator for PKM2 that results in the shunting of glycolytic intermediates away from serine biosynthesis pathways, rendering cancer cells auxotrophic toward serine. This demonstrates that auxotrophism may represent an evolvable trait in cancer cells.

Importantly, although auxotrophism is usually described with regards to metabolic substrates, the same concept may be applied to signaling molecules. As an example, the growth promoting effect of estrogen on estrogen receptor-positive tumors, as well as the “oncogene addiction” exhibited by some cancers (43) may be seen as a form of “tropism” where a signal instead of a metabolite is required for normal growth. These circuits (metabolic pathways or cell signaling cascades) may also overlap. As an example, the proto-oncogene c-Myc not only plays an important role in activating mitogenic signals but also plays an important role in shifting the metabolism of cancer cells toward aerobic glycolysis (44).

**Figure 2:**
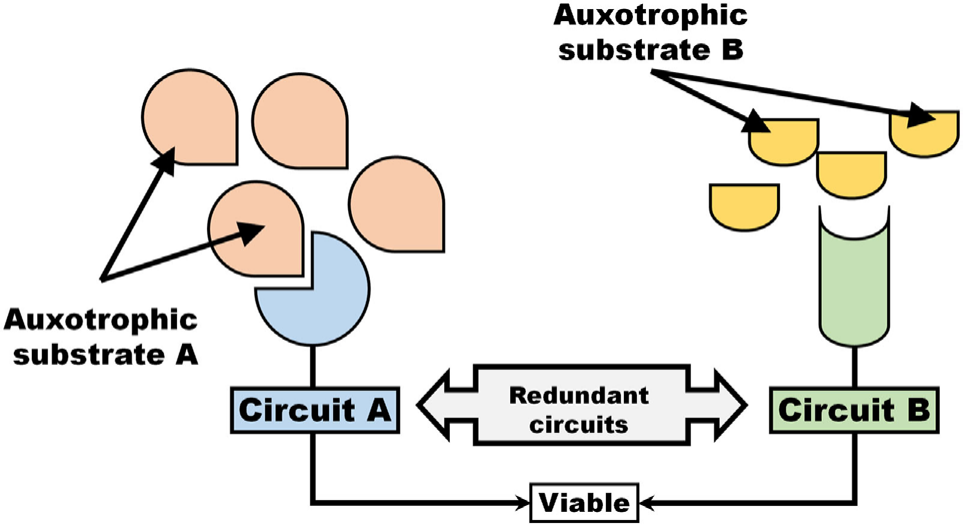
An auxotrophic system exhibits traits associated with synthetic lethality. Two pathways, Circuit A and Circuit B that operate independent and redundantly of each other, are dependent on auxotrophic factors (e.g., signaling molecule or metabolic substrate).

Induction of an auxotrophic phenotype can, if two circuits (e.g., metabolic pathways or signaling cascades) are redundant (i.e., once circuit could compensate for the loss of another), exhibit traits similar to synthetic lethality (Figure 2). As an example, losing the ability to synthesize a metabolite would render cells synthetically lethal toward the inhibition of a transporter (or withdrawal of auxotrophic factor). Similar to the description provided to synthetically lethal genes, two circuits A and B (Figure 2) can be considered where the inactivation of one or the other, but not both simultaneously, remains viable. Thus, evolution of an auxotrophic phenotype can occasionally represent as a form of synthetic lethality.

## Induction of Auxotrophic Phenotypes

**Figure 3:**
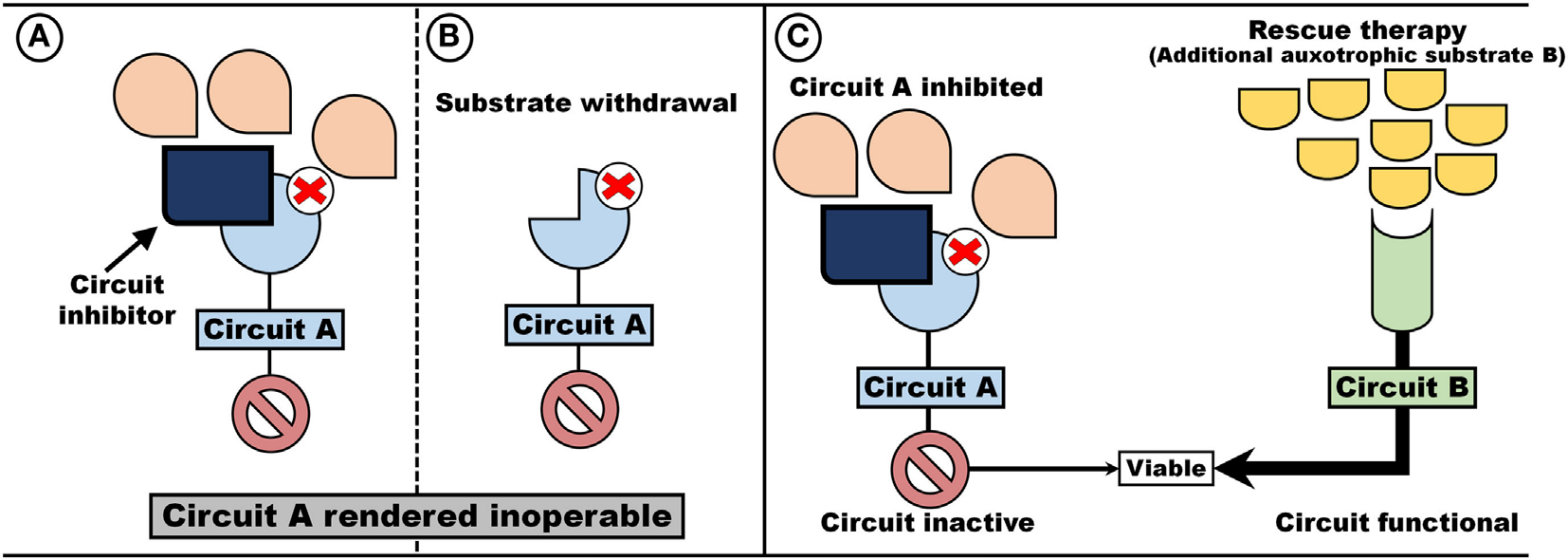
Auxotrophism can be artificially induced. Two pathways, Circuit A and Circuit B, which operate independent and redundant of each other, are dependent on auxotrophic factors (e.g., signaling molecules or metabolic substrates). By removing the substrate **(B)** or administering Circuit A blockers **(A)**, a cell become auxotrophic toward auxotrophic factors for Circuit B **(C)**.

An auxotrophic system with synthetic lethal attributes exhibits two unique properties (Figure 3). First, the value of maintaining a circuit (either A or, B, or both) can be controlled by rendering a circuit inoperable and thus remove the incentive for maintaining the circuit. As an example, either blocking the receptor/transporter (Figure 3A) or withdrawal of the auxotrophic factor (Figure 3B) would render the circuit non-functional and thus dispensable. Second, an auxotrophic system provides the opportunity to introduce “rescue therapy”: inactivation of one circuit may be compensated for by supplying an auxotrophic substrate for the compensating circuit (Figure 3C).

Such a hypothetical system represents a prime target for inducing an auxotrophic dependency in cancer cells. First, the selection regime is inducible by either limiting the auxotrophic factor (Figure 3B) or through the implementation of inhibitors (Figure 3A) (e.g., blocking a signaling cascade or limiting the import of a metabolic substrate). Since the circuit inhibited is of no value, loss of function mutation can occur without out loss of viability. Indeed, if maintenance of the inhibited circuit is associated with a cost, such a trait is more likely to be lost. Second, since circuits are redundant, on-target toxicity should be minimal and can theoretically be further decreased by the administration of “rescue therapy”—auxotrophic substrate utilized by the compensating circuit (Figure 3C).

**Figure 4:**
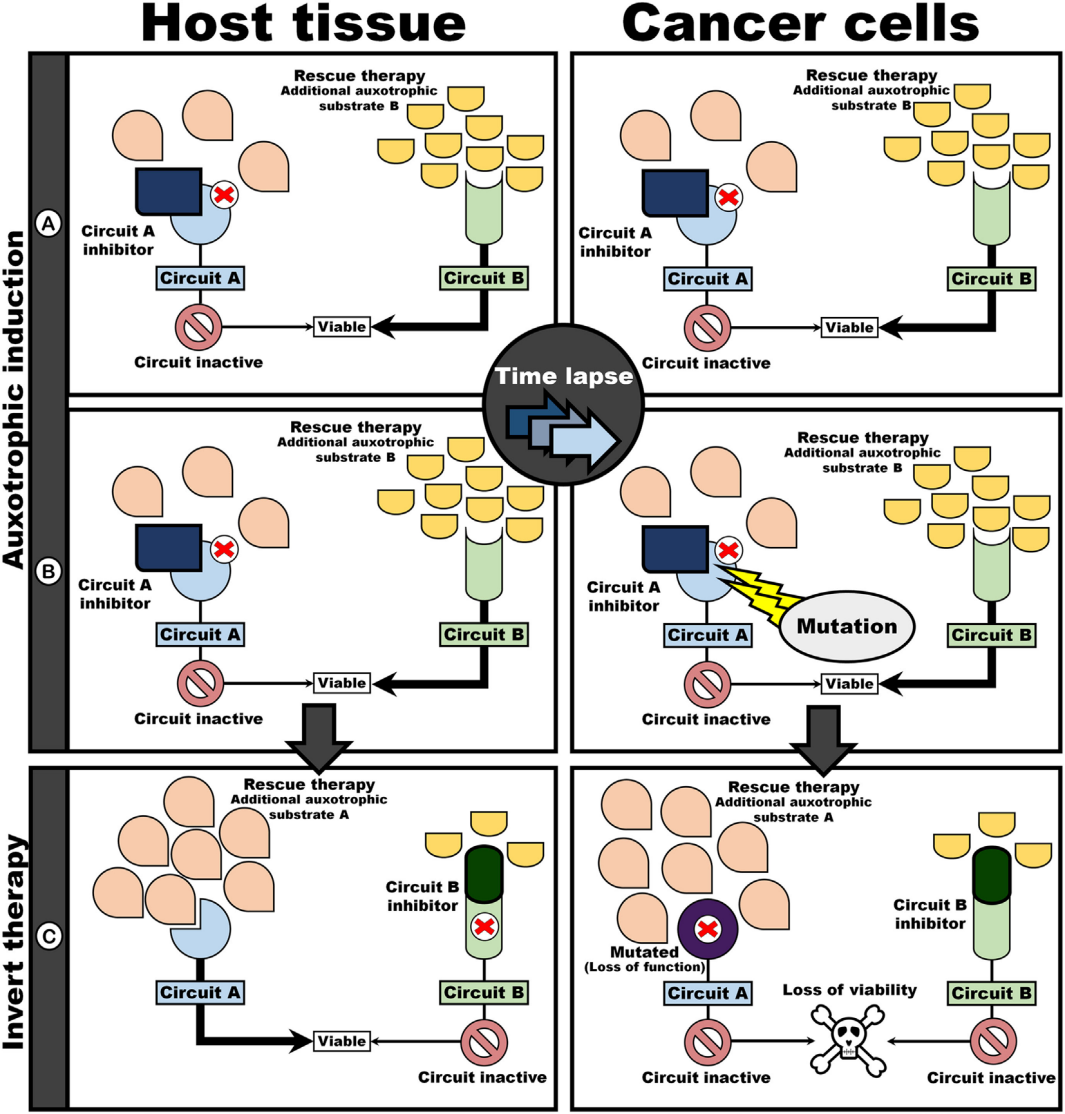
Evolutionary procedure for auxotrophic induction in cancer cells provides differential targets. Auxotrophy in cancer cells toward B is induced by simultaneously administering an inhibitor of A and an auxotrophic substrate for circuit B as rescue therapy **(A,B)**. Since maintenance of contextually useless circuits are expensive, cancer cell losing A would have a fitness advantage above cells maintaining A. Thus, sub-clones auxotrophically dependent on B would dominate. An intervention challenge would consist of inverting the “poison” and “antidote” regime **(C)**: inhibition of Circuit B while upregulating flux through Circuit A with an additional auxotrophic substrate. Mutated cancer cells are unresponsive to rescue by the auxotrophic substrate for A as a result in loss of function in circuit A. Thus, cancer cells no longer have a functioning circuit, resulting in cell death.

As a clinical application, i.e., where both host and cancer are subjected to the same intervention, the two-step approach will result in a therapeutic target unique to cancer cells (Figure 4). First, the initiation of an auxotrophic induction regime (AIR) is introduced (Figures 4A,B), with the goal of directing the evolution of auxotrophism in cancer cells. This is followed by therapeutic intervention, directed at targeting auxotrophic dependency in cancer cells (Figure 4C). The AIR consists of blocking circuit A, while simultaneously administering rescue therapy in the form of an additional auxotrophic substrate to compensate for Circuit B (Figure 4A). The inhibition of A would result in a viable phenotype as B can compensate for an inoperable A. That is, assuming perfect redundancy between A and B, both host and cancer cells would be viable under these conditions. However, since inhibition of Circuit A would render the circuit redundant, a mutation that knockout A in a cancer cell may benefit cancer cells if maintenance of A decrease replication efficiency (Figure 4C). Thus, a sub-clone of cancer cells that do not invest in the “contextually useless” A, but rather invest in B, would have an evolutionary advantage over cells that maintain A. However, such a beneficial mutation would render cells auxotrophic toward the substrate required to maintain circuit B (i.e., render cells dependent on auxotrophic substrate for circuit B). Following the induction of an autotropic phenotype, intervention therapy can now be implemented (Figure 4C) to exploit this auxotrophic dependency: by now inhibiting B, but administering rescue therapy in the form of a compensatory auxotrophic substrate for circuit A, host cells can compensate and remain viable. In contrast, cancer cells, now lacking a compensatory circuit A, are no longer viable.

In this manner, the evolvability of cancer cells can be turned against themselves: these “evolutionary successful” cells that lack circuit A are now auxotrophic toward substrate required for system B. Note that, because A and B are redundant, on-target toxicity to host and cancer cells would be minimum under the AIR. After AIR, intervention therapy can be initiated, by blocking B, while administering additional substrate for A (i.e., swapping the “poison” and “antidote” used in the AIR regime). Since host cells are genomically stable, these cells can reactivate A and remain viable despite B being pharmacologically knocked-out.

## Future Challenges

Biological systems are likely to exhibit various circuits (cell signaling cascades or metabolic pathways). Indeed, degeneracy, a “ubiquitous biological property” refers to the ability of a biological system to enact the same phenotype through different mechanisms (45). Thus, it is likely that various synthetically lethal circuits may be operating in cells, with the potential for auxotrophic induction. In addition, viewing signaling molecules as auxotrophic factors may widen the range of potential targets. Of key importance, however, would be to identify a candidate system that is costly to maintain: the higher the maintenance cost, the greater the evolutionary incentive to disinvest in the particular circuit (i.e., lose functionality). Also, if auxotrophic factors are secreted by other cells, it would render substrate/signaling withdrawal impossible. Alternately, serum levels of secreted auxotrophic targets would need to be reduced by administering enzymes capable of metabolizing these compounds or be inactivated/removed by chelators. Most likely, inhibitors or transporters could prove most useful.

Signaling pathways pose an additional challenge for auxotrophic induction, since cancer cells are more likely to evolve independence from exogenous signals (e.g., mutation of genes resulting in constitutively activated proteins). None the less, targeting signaling pathways may augment auxotrophic phenotypes since metabolic and signaling circuits are often intimately linked. As an example, *Kras* driver mutations regulate the metabolic reprogramming in pancreatic tumors (46). Similarly, activated EGFR induces translocation of PKM2 into the nucleus where it interacts with other transcription factors promoting tumor growth (47). HIF-1 also upregulates PKM2, while PKM2 acts as a cofactor of HIF-1, thus modulating its transcriptional activity (48). Collectively, these observations suggest that targeting signaling and metabolic circuits may provide synergistic effects in placing cancer cells on evolutionary trajectories. Alternatively, the set of oncogenic signaling profiles might highlight putative metabolic pathways, which might be best to target, as the signaling pathways might dictate the metabolic dependency of the cell.

An additional requirement for auxotrophic induction is that the redundancy in circuits must be substantial to allow for effective compensation by alternative pathways, thus minimize on-target toxicity. The requirement of redundancy is of particular importance as evolutionary therapies are likely to be implemented over an extended period of time. Also, evolutionary pressures select for phenotypes. Since a similar phenotype can be induced by various means, great care must be taken to ensure that the given selective regimes would result in the desired set of mutations. In this regard, network analysis of metabolic/signaling pathways may assist in identifying targetable circuits. Furthermore, it might be useful to target genes that are more likely to mutate, as it has been noted that regional mutation rates are not constant across the genomes of cancer cells (49). In particular, somatic mutation rates are highest in inaccessible, heterochromatin-like regions with low gene expression (49). This suggests that inactivated genes are more likely to undergo random mutations.

Another key challenge relate to the fact that cancer consists of heterogeneous populations of cells bearing different mutational profiles and, as such, are likely to respond differently to a particular selective pressure. Thus, it is exceedingly likely that all cells will evolve a targetable auxotrophic phenotype. Indeed, therapeutic failure, seen with conventional as well as with targeted therapy, suggests this is to be expected. Here, similar to current clinical practice with conventional therapeutic regimes, combination therapy can be implemented: cells can be placed on various selection regimes to induce multiple auxotrophisms simultaneously to maximize the possibility to target most cells. Similarly, “second-line auxotrophism selection” may be initiated as sequential therapy. In fact, because AIRs exhibit the potential to have reduced toxicities, it is possible that sequential cycling through various AIRs may be well tolerated for prolonged periods, resulting in a novel form of maintenance therapy.

It also remains to be established if the evolution of cancer takes place within a feasible time window. Cancer often evolves resistance to different therapeutic interventions within a matter of months, suggesting that the evolution of cancer can be rapid. However, although chemotherapy represents a very strong selective pressure, it is not obvious whether cancer cells can be subjected to a strong enough selection regime to similarly evolve an auxotrophic phenotype within a realistic time window. These considerations also suggest that certain cancers, such as those exhibiting a mutator phenotype, may be more amenable to this strategy. Also, evolution of an auxotrophic phenotype is much more likely if an auxotrophic clone exhibits a competitive advantage over non-auxotrophic clones. As an example, if inhibition of a metabolic pathway results in the buildup of a potentially toxic metabolic intermediate, auxotrophic clones that do not invest in the metabolic pathway (i.e., lack the biosynthetic ability) would have a competitive advantage since they would avoid accumulating toxic intermediates. An example of such a process is the loss of vitamin C synthesis in animals that acquire sufficient amounts of vitamin C through their diet, which is argued to develop, since loss of synthetic capacity would avoid the generation of hydrogen peroxide, which occurs during vitamin C synthesis (36). In addition, many patients enter remission for years after initial chemotherapy. However, the sad reality is that for many of these patients, tumors do reoccur. Here, since an AIR may have very low toxicity, incorporating a prophylactic induction regimes in the survivorship care plan may be beneficial: therefore, if cancer is predestined to re-emerge, the cancer cells will expand under a pre-existing selective pressure. This would have the effect of a “population bottle-neck” and also extend the time period under which cancer cells are subjected to selective pressures. This would enhance the likelihood of cancer evolving an auxotrophic phenotype within a realistic time frame.

**Figure 5:**
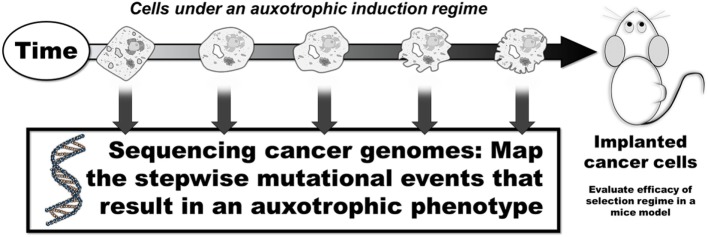
Mapping the stepwise mutational development of auxotrophic phenotypes. *In vitro* selection regimes may result in the development of auxotrophic phenotypes. Mapping the stepwise genomic changes may provide insight into the evolutionary steps that induce an auxotrophic phenotype.

Another key question not addressed is which particular molecular circuits should be targeted and can these circuits be targeted with minimum toxicity. Also, can the evolution of cancer be effectively predicted? Here, as proof of concept, initial studies may subject cells to a selection regime to induce an auxotrophism *in vitro*. The “evolved” (i.e., auxotrophic) cancer cells can then be injected into a mouse model, and the intervention therapy be applied to test the efficacy of the selection regime. It may also be useful to investigate how evolution of a phenotype manifests. In this regard, genotyping cancer cells under selection for an auxotrophic phenotype may be instructive: successfully mapping the genomic alteration that drive the auxotrophic phenotype may be valuable in developing models that predict the evolution of cancer cells to refine the selection regimes (Figure 5). A subsequent step may include exploring the feasibility of this approach in companion animals in comparative oncology trials (50). Here, the clinical application of auxotrophic phenotypes (under auxotrophic regimes that exhibit minimum toxicity) may be applied concurrently with standard therapy, or as mentioned, initiated during remission to test the feasibility of this hypothesis in a more realistic setting before applying the therapy in humans.

## Conclusion

The low toxicity of this approach may suggest that auxotrophic induction can provide effective adjuvant therapy. In settings where curative therapy is no longer realistic, cycling through different evolutionary regimes (i.e., inducing various auxotrophisms sequentially) may provide a novel form of maintenance therapy. We also suspect that phenotypes other than synthetic lethality could be selected for in cancer. As an example, cancer cells could be selected for a less noxious phenotype.

## Author Contributions

GN, TN, and A-ME conceived, drafted, and revised the manuscript. All authors read and approved the final manuscript.

## Conflict of Interest Statement

The authors declare that the research was conducted in the absence of any commercial or financial relationships that could be construed as a potential conflict of interest.

## Abbreviations

AIR, auxotrophic induction regime.

## References

[1] Wan L Pantel K Kang Y. Tumor metastasis: moving new biological insights into the clinic. Nat Med (2013) 19:1450–64. 10.1038/nm.3391 24202397

[2] Turner NC Reis-Filho JS. Genetic heterogeneity and cancer drug resistance. Lancet Oncol (2012) 13:e178–85. 10.1016/S1470-2045(11)70335-7 22469128

[3] Bock C Lengauer T. Managing drug resistance in cancer: lessons from HIV therapy. Nat Rev Cancer (2012) 12:494–501. 10.1038/nrc3297 22673150

[4] Lee AJ Swanton C. Tumour heterogeneity and drug resistance: personalising cancer medicine through functional genomics. Biochem Pharmacol (2012) 83:1013–20. 10.1016/j.bcp.2011.12.008 22192819

[5] Gillies RJ Verduzco D Gatenby RA. Evolutionary dynamics of carcinogenesis and why targeted therapy does not work. Nat Rev Cancer (2012) 12:487–93. 10.1038/nrc3298 22695393 PMC4122506

[6] Hanahan D Rethinking the war on cancer. Lancet (2014) 383:558–63. 10.1016/S0140-6736(13)62226-6 24351321

[7] Goldstein I Madar S Rotter V. Cancer research, a field on the verge of a paradigm shift? Trends Mol Med (2012) 18:299–303. 10.1016/j.molmed.2012.04.002 22609171

[8] Gatenby RA Gillies RJ Brown JS. Of cancer and cave fish. Nat Rev Cancer (2011) 11:237–8. 10.1038/nrc3036 21548400 PMC3971416

[9] Merlo LM Pepper JW Reid BJ Maley CC. Cancer as an evolutionary and ecological process. Nat Rev Cancer (2006) 6:924–35. 10.1038/nrc2013 17109012

[10] Aktipis CA Nesse RM. Evolutionary foundations for cancer biology. Evol Appl (2013) 6:144–59. 10.1111/eva.12034 23396885 PMC3567479

[11] Gatenby RA Brown J Vincent T. Lessons from applied ecology: cancer control using an evolutionary double bind. Cancer Res (2009) 69:7499–502. 10.1158/0008-5472.CAN-09-1354 19752088

[12] Pluchino KM Hall MD Goldsborough AS Callaghan R Gottesman MM Collateral sensitivity as a strategy against cancer multidrug resistance. Drug Resist Updat (2012) 15:98–105. 10.1016/j.drup.2012.03.002 22483810 PMC3348266

[13] Saunders NA Simpson F Thompson EW Hill MM Endo-Munoz L Leggatt G Role of intratumoural heterogeneity in cancer drug resistance: molecular and clinical perspectives. EMBO Mol Med (2012) 4:675–84. 10.1002/emmm.201101131 22733553 PMC3494067

[14] Sidow A Spies N. Concepts in solid tumor evolution. Trends Genet (2015) 31:208–14. 10.1016/j.tig.2015.02.001 25733351 PMC4380537

[15] Tam WL Weinberg RA. The epigenetics of epithelial-mesenchymal plasticity in cancer. Nat Med (2013) 19:1438–49. 10.1038/nm.3336 24202396 PMC4190672

[16] Marusyk A Almendro V Polyak K. Intra-tumour heterogeneity: a looking glass for cancer? Nat Rev Cancer (2012) 12:323–34. 10.1038/nrc3261 22513401

[17] Greenman C Stephens P Smith R Dalgliesh GL Hunter C Bignell G Patterns of somatic mutation in human cancer genomes. Nature (2007) 446:153–8. 10.1038/nature05610 17344846 PMC2712719

[18] Merlo LM Maley CC The role of genetic diversity in cancer. J Clin Invest (2010) 120:401 10.1172/JCI42088 20101092 PMC2810093

[19] Gerlinger M Rowan AJ Horswell S Larkin J Endesfelder D Gronroos E Intratumor heterogeneity and branched evolution revealed by multiregion sequencing. N Engl J Med (2012) 366:883–92. 10.1056/NEJMoa1113205 22397650 PMC4878653

[20] Loeb LA Springgate CF Battula N Errors in DNA replication as a basis of malignant changes. Cancer Res (1974) 34:2311–21. 4136142

[21] Loeb LA. Human cancers express mutator phenotypes: origin, consequences and targeting. Nat Rev Cancer (2011) 11:450–7. 10.1038/nrc3063 21593786 PMC4007007

[22] Duesberg P Stindl R Hehlmann R. Explaining the high mutation rates of cancer cells to drug and multidrug resistance by chromosome reassortments that are catalyzed by aneuploidy. Proc Natl Acad Sci U S A (2000) 97:14295–300. 10.1073/pnas.97.26.14295 11121035 PMC18912

[23] van Gent DC Kanaar R. Exploiting DNA repair defects for novel cancer therapies. Mol Biol Cell (2016) 27:2145–8. 10.1091/mbc.E15-10-0698 27418635 PMC4945134

[24] Benedict WF Baker MS Haroun L Choi E Ames BN. Mutagenicity of cancer chemotherapeutic agents in the *Salmonella*/microsome test. Cancer Res (1977) 37:2209–13. 193638

[25] Pigliucci M. Is evolvability evolvable? Nat Rev Genet (2008) 9:75–82. 10.1038/nrg2278 18059367

[26] Chang HY Sneddon JB Alizadeh AA Sood R West RB Montgomery K Gene expression signature of fibroblast serum response predicts human cancer progression: similarities between tumors and wounds. PLoS Biol (2004) 2:E7 10.1371/journal.pbio.0020007 14737219 PMC314300

[27] Rousseaux S Debernardi A Jacquiau B Vitte AL Vesin A Nagy-Mignotte H Ectopic activation of germline and placental genes identifies aggressive metastasis-prone lung cancers. Sci Transl Med (2013) 5:186ra66. 10.1126/scitranslmed.3005723 23698379 PMC4818008

[28] Louwen F Muschol-Steinmetz C Reinhard J Reitter A Yuan J. A lesson for cancer research: placental microarray gene analysis in preeclampsia. Oncotarget (2012) 3:759–73. 10.18632/oncotarget.595 22929622 PMC3478454

[29] Arnal A Ujvari B Crespi B Gatenby RA Tissot T Vittecoq M Evolutionary perspective of cancer: myth, metaphors, and reality. Evol Appl (2015) 8:541–4. 10.1111/eva.12265 26136820 PMC4479510

[30] Lawrence MS Stojanov P Polak P Kryukov GV Cibulskis K Sivachenko A Mutational heterogeneity in cancer and the search for new cancer-associated genes. Nature (2013) 499:214–8. 10.1038/nature12213 23770567 PMC3919509

[31] Kaelin WG. The concept of synthetic lethality in the context of anticancer therapy. Nat Rev Cancer (2005) 5:689–98. 10.1038/nrc1691 16110319

[32] Nijman S. Synthetic lethality: general principles, utility and detection using genetic screens in human cells. FEBS Lett (2011) 585:1–6. 10.1016/j.febslet.2010.11.024 21094158 PMC3018572

[33] Wang T Yu H Hughes NW Liu B Kendirli A Klein K Gene essentiality profiling reveals gene networks and synthetic lethal interactions with oncogenic Ras. Cell (2017) 168:890–903.e15. 10.1016/j.cell.2017.01.013 28162770 PMC5445660

[34] Lord CJ Ashworth A. PARP inhibitors: synthetic lethality in the clinic. Science (2017) 355:1152–8. 10.1126/science.aam7344 28302823 PMC6175050

[35] Drouin G Godin JR Page B. The genetics of vitamin C loss in vertebrates. Curr Genomics (2011) 12:371–8. 10.2174/138920211796429736 22294879 PMC3145266

[36] Bánhegyi G Csala M Braun L Garzó T Mandl J. Ascorbate synthesis-dependent glutathione consumption in mouse liver. FEBS Lett (1996) 381:39–41. 10.1016/0014-5793(96)00077-4 8641435

[37] Helliwell KE Wheeler GL Smith AG. Widespread decay of vitamin-related pathways: coincidence or consequence? Trends Genet (2013) 29:469–78. 10.1016/j.tig.2013.03.003 23623319

[38] Feun L You M Wu CJ Kuo MT Wangpaichitr M Spector S Arginine deprivation as a targeted therapy for cancer. Curr Pharm Des (2008) 14:1049–57. 10.2174/138161208784246199 18473854 PMC3096551

[39] Qiu F Huang J Sui M. Targeting arginine metabolism pathway to treat arginine-dependent cancers. Cancer Lett (2015) 364:1–7. 10.1016/j.canlet.2015.04.020 25917076

[40] Changou CA Chen YR Xing L Yen Y Chuang FY Cheng RH Arginine starvation-associated atypical cellular death involves mitochondrial dysfunction, nuclear DNA leakage, and chromatin autophagy. Proc Natl Acad Sci U S A (2014) 111:14147–52. 10.1073/pnas.1404171111 25122679 PMC4191793

[41] Szlosarek PW Steele JP Nolan L Gilligan D Taylor P Spicer J Arginine deprivation with pegylated arginine deiminase in patients with argininosuccinate synthetase 1-deficient malignant pleural mesothelioma: a randomized clinical trial. JAMA Oncol (2017) 3:58–66. 10.1001/jamaoncol.2016.3049 27584578

[42] Kung C Hixon J Choe S Marks K Gross S Murphy E Small molecule activation of PKM2 in cancer cells induces serine auxotrophy. Chem Biol (2012) 19:1187–98. 10.1016/j.chembiol.2012.07.021 22999886 PMC3775715

[43] Weinstein IB Joe A Oncogene addiction. Cancer Res (2008) 68:3077–80; discussion 3080. 10.1158/0008-5472.CAN-07-3293 18451130

[44] Dang CV c-Myc target genes involved in cell growth, apoptosis, and metabolism. Mol Cell Biol (1999) 19:1–11. 10.1128/MCB.19.1.1 9858526 PMC83860

[45] Edelman GM Gally JA. Degeneracy and complexity in biological systems. Proc Natl Acad Sci U S A (2001) 98:13763–8. 10.1073/pnas.231499798 11698650 PMC61115

[46] Ying H Kimmelman AC Lyssiotis CA Hua S Chu GC Fletcher-Sananikone E Oncogenic Kras maintains pancreatic tumors through regulation of anabolic glucose metabolism. Cell (2012) 149:656–70. 10.1016/j.cell.2012.01.058 22541435 PMC3472002

[47] Yang W Xia Y Ji H Zheng Y Liang J Huang W Nuclear PKM2 regulates [bgr]-catenin transactivation upon EGFR activation. Nature (2011) 480:118–22. 10.1038/nature10598 22056988 PMC3235705

[48] Luo W Hu H Chang R Zhong J Knabel M O’Meally R Pyruvate kinase M2 is a PHD3-stimulated coactivator for hypoxia-inducible factor 1. Cell (2011) 145:732–44. 10.1016/j.cell.2011.03.054 21620138 PMC3130564

[49] Schuster-Böckler B Lehner B Chromatin organization is a major influence on regional mutation rates in human cancer cells. Nature (2012) 488:504–7. 10.1038/nature11273 22820252

[50] Gordon I Paoloni M Mazcko C Khanna C The comparative oncology trials consortium: using spontaneously occurring cancers in dogs to inform the cancer drug development pathway. PLoS Med (2009) 6:e1000161 10.1371/journal.pmed.1000161 19823573 PMC2753665

